# The role of RNF149 in the pre-emptive quality control substrate ubiquitination

**DOI:** 10.1038/s42003-023-04763-9

**Published:** 2023-04-08

**Authors:** Aster Legesse, Nathali Kaushansky, Ilana Braunstein, Haddas Saad, Gerardo Lederkremer, Ami Navon, Ariel Stanhill

**Affiliations:** 1 Department of Natural and Life Sciences, Open University of Israel, Ra’anana, 43710 Israel; 2 Department of Molecular Cell Biology, Weizmann institute of Science, Rehovot, 7610001 Israel; 3 Department of Biochemistry, Technion School of Medicine, Haifa, 31096 Israel; 4 The Shmunis School of Biomedicine and Cancer Research, George Wise Faculty of Life Sciences, Tel-Aviv University, Tel-Aviv, 69978 Israel; 5 Department of Immunology and Regenerative Biology, Weizmann institute of Science, Rehovot, 7610001 Israel

**Keywords:** ER-associated degradation, Endoplasmic reticulum

## Abstract

Protein quality control is a process in which a protein’s folding status is constantly monitored. Mislocalized proteins (MLP), are processed by the various quality control pathways, as they are often misfolded due to inappropriate cellular surroundings. Polypeptides that fail to translocate into the ER due to an inefficient signal peptide, mutations or ER stress are recognized by the pre-emptive ER associated quality control (pEQC) pathway and degraded by the 26 S proteasome. In this report we reveal the role of RNF149, a membrane bound E3 ligase in the ubiquitination of known pEQC substrates. We demonstrate its selective binding only to non-translocated proteins and its association with known pEQC components. Impairment in RNF149 function increases translocation flux into the ER and manifests in a myeloproliferative neoplasm (MPN) phenotype, a pathological condition associated with pEQC impairment. Finally, the dynamic localization of RNF149 may provide a molecular switch to regulate pEQC during ER stress.

## Abstract (web summary)

E3 ligase RNF149 contributes to the pre-emptive quality control (pQC) pathway & interacts with AIRAPL in a ubiquitin-dependent manner, with loss of RNF149 function appearing to increase the translocation flux of pQC substrates to the ER.

## Introduction

The cellular response to protein misfolding is a continuous challenge the cell must undertake in order to maintain protein functionality in a dynamic surrounding. While specific cellular pathways have evolved to deal with general and organelle specific stress responses (ER, peroxisome, nuclear, mitochondrial and Golgi), the unfolded protein response (UPR) probably represents the most comprehensive organelle response. In recent years it has become evident that regulation of ER translocation during stress can be viewed as an additional UPR arm^1–3^, that in-concert with gene expression reprograming^4,5^, RNA stability^6^ and translational regulation^7^, enable the ER to cope with the inflicted stress. In addition to stress regulated translocation, protein targeting and successful translocation into the ER is a complex and imperfect process that gives rise to mislocalized proteins (MLP). These proteins are often misfolded, interact promiscuously with surrounding proteins and are highly toxic^8,9^. As such, the surveillance on ER translocation must be finely balanced between successful translocation of versatile polypeptides and effective and quick elimination of MLP that are failing to translocate. Another layer of complexity to these processes rises from the fact that MLP from the ER stem from two independent processes of ER quality surveillance. One is the translocation into the ER (the pre-emptive ER associated quality control; pEQC)^2^ and the other is retro-translocation (ER associated degradation; ERAD)^10,11^. Several components of these protein quality machineries participate in more than one process. Translocation of tail anchored proteins is mediated by a Bag6 holdase complex^12,13^ as well as its participation in ERAD and the pEQC process^14,15^. In this case the interplay between the various Bag6 partners, enables Bag6 to triage proteins between translocation and elimination^16^. Similarly, p97 association with various co-factors enable it to participate in ribosome quality control, ERAD and pEQC processes^1,10,11,17,18^. Thus, the interplay between the specific and general factors presumably enable to coordinate and fine-tune the fate of MLP by the various pathways.

## Results

### RNF149 binding to pEQC components

**Fig. 1:**
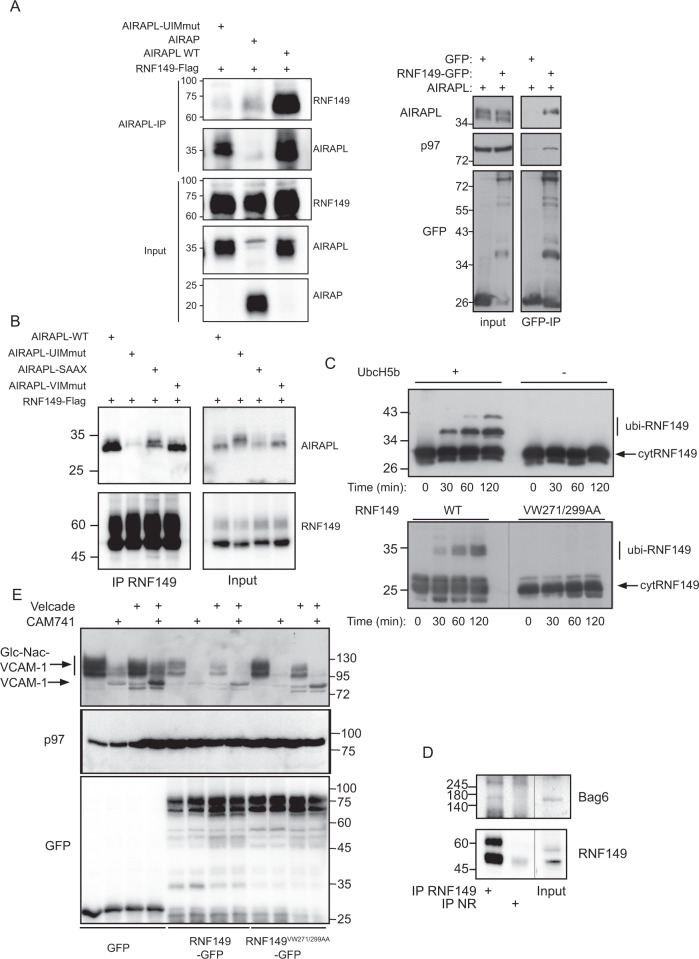
RNF149 binding to pEQC components. **A** Left-293 cell lysates expressing RNF149-Flag alongside the indicated AIRAPL isoform or AIRAP (used as a specificity control), were immunopurified (IP) with AIRAP or AIRAPL antibodies and RNF149 content was revealed with a Flag immunoblot. Total content prior to IP is indicated as input. Right- 293 cell lysates expressing AIRAPL alongside the indicated RNF149-GFP or GFP (used as a specificity control), were IPed with a GFP antibody. AIRAPL and endogenous p97 content were revealed by immunoblot. Total content prior to IP is indicated as input. **B** 293 cell lysates expressing RNF149 alongside the indicated AIRAPL isoforms were IPed with an RNF149 antibody. AIRAPL content was revealed by immunoblot. Total content prior to IP is indicated as input. **C** A time course in-vitro ubiquitin ligase assay using recombinant cytosolic portion of RNF149 (AA222-400) as a E3 ligase, in the presence or absence of the E2 UbcH5b (upper panel) or a RING mutant VW271/299AA (lower panel). **D** Binding of endogenous RNF149 to endogenous Bag6 was evaluated by an IP of RNF149 or a non-relevant (N.R.) antibody IP from 293 cells stably expressing the pEQC substrate VCAM1-YFP. Bag6 content was revealed by immunoblot. Total content prior to IP is indicated as input. **E** Cells expressing VCAM-1 alongside GFP, RNF149WT-GFP or RNF149RINGmut-GFP were treated with CAM741 or velcade as indicated. The presence of the non-translocated (VCAM-1) and glycosylated translocated protein (Glc-NAC-VCAM-1) are indicated. GFP immunoblot show the expression level of the free or fused RNF149 forms and p97 IB serves as a loading control of lysates.

Previous biochemical purifications have identified arsenite-inducible RNA-associated protein-like (AIRAPL; Zfand2b) as a p97 cofactor with specificity towards K48 based poly-ubiquitin chains^19,20^. During this purification RNF149, a putative E3 ligase was found to co-purify with AIRAPL^19^. RNF149 domain architecture predicts RNF149 to be a type I transmembrane domain protein with an N-terminal signal-peptide, protease associated (PA) domain, a short 20 amino acid helical membrane stretch and a cytosolic RING domain. To confirm this interaction and to better characterize it we performed co-immunopurification (IP) of AIRAPL and monitored the association of RNF149 in the purified complex. The RNF149-AIRAPL interaction is highly dependent on the ability of AIRAPL to bind ubiquitin as a non-ubiquitin interacting motif (UIM) mutant of AIRAPL (UIMmut^19^) showed diminished ability to bind RNF149. Furthermore, a similar gene that does not bind ubiquitin (AIRAP;Zfand2a), was also impaired in binding RNF149 (Fig. 1A-left panel). Reciprocal purifications of RNF149 confirmed specificity of RNF149 interaction with AIRAPL as a cofactor complex with p97 (Fig. 1A-right panel). Further analysis of AIRAPL mutations that may impair RNF149 binding include a membrane binding mutant, p97 binding mutant and the ubiquitin binding mutant^19–21^. Co-purification of the various AIRAPL mutants by RNF149 IP confirmed the role for ubiquitin binding (UIM mutant) but not of p97 binding in RNF149 interaction., as the AIRAPL VCP interacting motif (VIM) mutant readily bound RNF149^19^ (Fig. 1B). An ER membrane mutant form of AIRAPL (no palmitoylation modification;AIRAPL SAAX;^21^) only partially reduced the binding of RNF149 to AIRAPL. Next, we evaluated the E3 ligase activity of RNF149 by means of an in-vitro ubiquitination assay (Fig. 1C) that confirmed E2 dependency as well as RING domain functionality, as a predicted RING mutant abolished this activity. In order to capture the endogenous interaction of RNF149 with pEQC factors such as Bag6^14^, we performed an immunoprecipitation of RNF149 from 293 cells that stably express a pEQC substrate (VCAM1-YFP; supplementary figure 1) in an attempt to increase the association of RNF149 with pEQC factors^22^. As seen in Fig. 1D, RNF149 association with Bag6 was detected upon RNF149 IP and not detected when a non-relevant (N.R.) IP was performed as a control. This result corroborates the expected association of RNF149 with pEQC factors involved in early events of substrate processing^14–16^.

The role of AIRAPL in the quality control of ER MLP^17,23^ and its association with RNF149 may place the E3 ligase in the proteasomal processing of mislocalized ER translocating proteins. To address such a possibility, we transfected into cells a pEQC substrate and evaluated the effect of RNF149 expression on the substrates steady state levels. VCAM-1 was chosen as the substrate because its translocation into the ER lumen is inhibited by a small molecule (CAM741) binding to Sec61^24^ and the non-glycosylated VCAM-1 can be readily distinguished based on the reduced mobility^17^; (Fig. 1A therein). As shown in Fig. 1E, VCAM-1 ER translocation inhibition is evident from the lack of HMW glycosylation pattern of VCAM-1 (Glc-Nac VCAM-1) and proteasomal inhibition (velcade) stabilized a unglycosylated form of non-translocated VCAM-1 (velcade+CAM741 conditions). Expression of RNF149 decreased the overall expression of VCAM-1 even in untreated cells attesting to the lack of efficient translocation of VCAM-1 even in the absence of CAM741. The inefficient translocation is also evident from the ability of velcade to stabilize a non-translocated form of VCAM-1 even in the absence of CAM741 (see LMW band in Fig. 1E lane 3). This effect of RNF149 on VCAM-1 translocation is completely dependent on its catalytic activity as no impact on VCAM-1 is noted upon expression of a RNF149 RING mutant (Fig. 1E).

### pEQC substrate association with RNF149

**Fig. 2:**
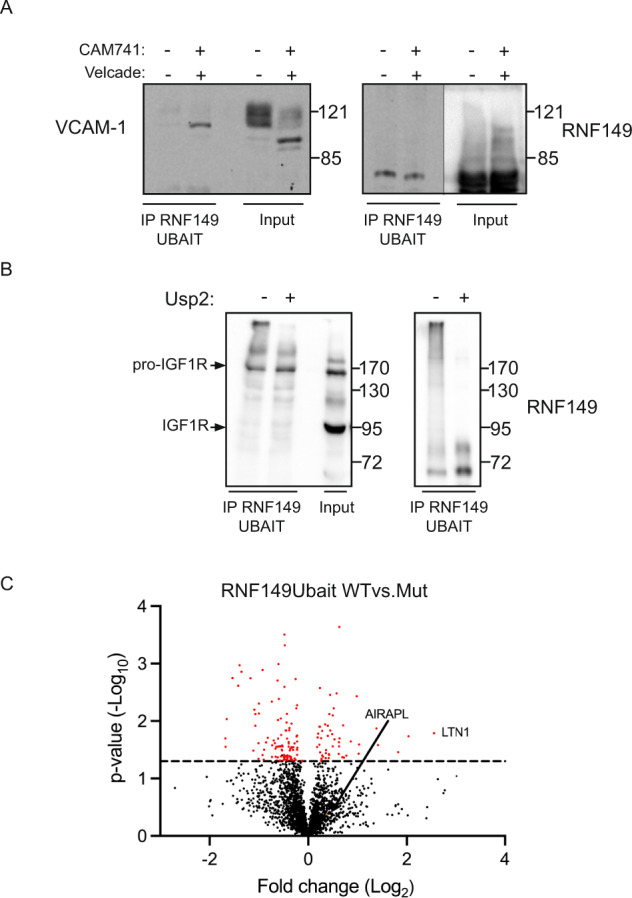
pEQC substrate association with RNF149. **A** 293 cells expressing RNF149UBAIT and the pEQC substrate VCAM-1 were treated as indicated with velcade and CAM741. Cell lysates were adjusted to RIPA buffer and a Flag IP was applied followed by extensive RIPA buffer washes. IP content was evaluated towards RNF149 and VCAM-1 content by Flag and HA immunoblots respectively. Total content prior to IP is indicated as input. **B** 293 cells expressing IGF1R-GFP and RNF149UBAIT were subjected to a Flag IP under RIPA conditions. Following extensive RIPA buffer washes the IP content was split into two equal fractions and the deubiquitinating enzyme Usp2 was added to half of the purified content. Deubiquitintion was terminated by addition of laemmli buffer and RNF149 and IGF1R content was evaluated by Flag (RNF149) and GFP (IGF-1R) immunoblots. **C** 293 cells expressing RNF149UBAIT wt or mutant forms were purified from cell extracts and protein content identification was evaluated by LC-MS. A volcano plot showing the enrichment fold (*X*-axis) and significance (*Y*-axis) is shown. In addition to AIRAPL, the top ranked specific interactor with RNF149UBAIT wt is labeled (LTN1).

To further address the specific role of RNF149 in processing of pEQC substrates, we reasoned that the interaction of the ligase should be detected only with the non-translocated mislocalized forms of a pEQC substrate and not with the translocated forms as they are not pEQC substrates. To allow the detection of E3 ligase substrates, ubiquitin fusion to a ligase enables to covalently bind the ligase with the substrates and to detect such transient interactions^25^. We expressed in cells a C-terminal ubiquitin fusion of RNF149 (RNF149UBAIT) and evaluated its ability to interact with VCAM-1 under control (ER translocated) or CAM741 + velcade (pEQC substrate) conditions. Cell lysates were immunoprecipitated to purify RNF149UBAIT and purified material was evaluated towards RNF149UBAIT and VCAM-1 content. While VCAM-1 was present in both glycosylated and non- glycosylated forms (Fig. 2A-input), only the non- glycosylated pEQC substrate form was present in the RNF149UBAIT IP (Fig. 2A IP). This result confirms the interaction of RNF149 only with mislocalized VCAM-1. We evaluated another pEQC substrate, IGF-1R, whose translocation into the ER has been shown to be regulated by AIRAPL^23^. Unlike VCAM-1, pro-IGF-1R (the non-processed αβ polypeptide) can be readily seen in the input material without CAM741 treatment (Fig. 2B-input). We wanted to evaluate if the interaction of RNF149 is specific to mislocalized IGF-1R (pro-IGF-1R) and not with IGF-1R. The evaluation of the RNF149UBAIT IP material did not detect any IGF-1R but only pro-IGF-1R (Fig. 2B-IP). Furthermore, performing an in-vitro de-ubiquitination assay on the purified material (Fig. 2B; + Usp2) revealed that RNF149UBAIT runs as a HMW smear that is caused by ubiquitination (Fig. 2B; IP RNF149 immunoblot). Thus, like AIRAPL, RNF149 interacts mainly with mislocalized pro-IGF1R and the minor species that are pEQC substrates (non-glycosylated VCAM-1 or pro-IGF-1R). We attribute the differences in appearance of RNF149UBAIT substrates (HMW polyubiquitinated smear with IGF-1R or unsmeared with VCAM-1), to differences in the interaction towards the two pEQC substrates, differences that may result from their translocation efficiencies. This would increase pro-IGF-1R interaction with RNF149, leading towards a more efficient polyubiquitination. To extend the specific evaluation of RNF149 interaction with potential substrates, we conducted a mass-spectrometry purification of wt and mutant RNF149UBAIT (containing the VW271/299AA mutation described previously) and compared their interaction profiles. We confirmed the interaction of RNF149 with AIRAPL alongside several components of the translocation machinery (Sec61, Sec62, Sec63, Sec11, Tram1) and several EMC components (EMC1-4, 6,8,10). However, these interactions were not specific to the RNF149UBAIT wt form. A specific RNF149UBAIT wt interaction was observed with the ribosome quality control (RQC) component (LTN1; Fig. 2C).

### RNF149 knockout phenotype and impact on translocation

**Fig. 3:**
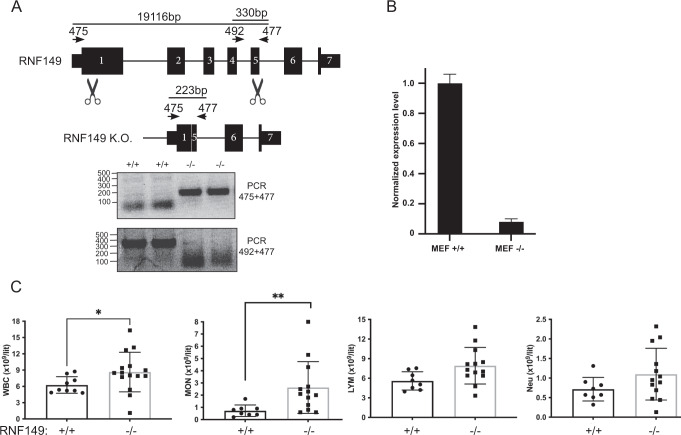
RNF149 knockout phenotype. **A** Mouse genomic locus illustration of *RNF149*. Forward and reverse primer binding sites (475, 492 and 477) are shown as well as scissor icons indicating the designed Cas9 cleavage sites. Expected PCR product size of the primer sets are indicated. Genotyping examples of RNF149 + / + and RNF149-/- animals using the designated primer sets shown on bottom. PCR products were sequenced and confirmed the deletion of the 19 kb RNF149 genomic fragment. **B** RT-PCR results of cDNA samples from the indicated genotypes were performed on MEF cells from the respective mice. RNA levels were normalized to a housekeeping gene (HPRT1). **C** Blood cells extracted from the indicated RNF149 genotype were evaluated at the age of 6 month. WBC-white blood cells; Neu-neutrophils; Mon-monocyte; Lym-lymphocyte. *p < 0.05; **p < 0.01.

**Fig. 4:**
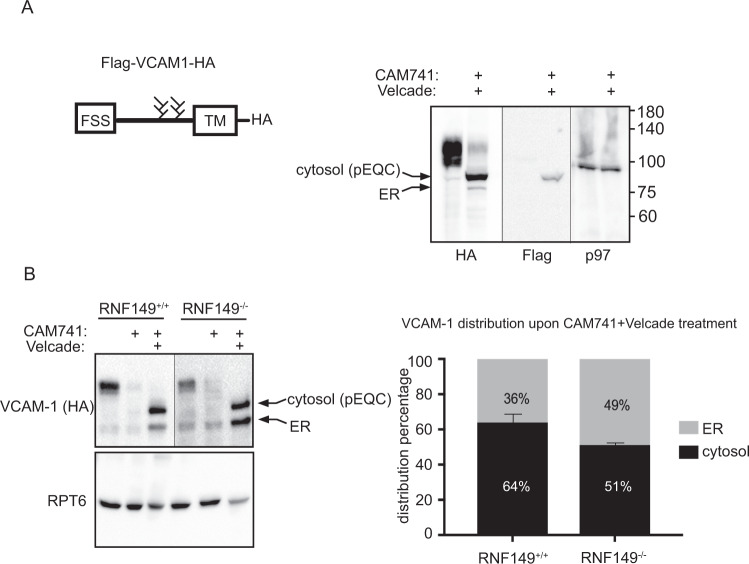
RNF149 knockout impact on translocation. **A** Left- Illustration of VCAM-1 containing the Flag signal sequence (FSS), the glycosylation sites, transmembrane domain (TM), and C-terminus HA tag. Right-Cells expressing Flag-VCAM1-HA were untreated or subjected to velcade and CAM741 treatment. The HA immunoblot reveals both ER and cytosol species of VCAM1 whereas the Flag immunoblot reveals only the pEQC non-translocated species that harbors the signal sequence. The lower band that is not recognized by the Flag immunoblot is an ER translocated species whose signal sequence has been cleaved. Proteasomal stabilization of this species (also seen in Fig. 1C with only velcade) indicates that a small percentage of VCAM1 is also subjected to ERAD. **B** RNF149 wildtype and knockout MEF cells stably expressing VCAM1 were treated with CAM741 and velcade as indicated. Steady state levels of VCAM1 were revealed by a HA immunoblot and ratios of pEQC or ERAD were quantified and standard deviations from three repetitions are presented.

To evaluate the in-vivo role of RNF149, we generated knockout mice by deleting a 19 kb genomic fragment encoding the PA, transmembrane and RING domains of RNF149 (Fig. 3A). R.T. PCR evaluation of RNF149 expression confirmed the reduced expression in the cells (Fig. 3B) and genomic sequencing confirmed the 19 kb deletion. pEQC impairment observed in AIRAPL knockout mice have a myeloproliferative neoplasms (MPN) phenotype and MPN patients were found to express reduced amounts of AIRAPL^23^. We therefore evaluated the proliferation status of hematological linage derived cells in the generated RNF149 knockout mice. As seen in Fig. 3C, hyperproliferation of several hematological linages was observed in the RNF149 knockout mice, demonstrating a similar phenotype with those observed in the AIRAPL knockout mice^23^. The deletion of RNF149 enabled to directly test the impact of RNF149 on pEQC substrate translocation, as the phenotypic observations of the AIRAPL knockout mice were explained by increased pEQC substrate translocation^23^. We therefore compared the translocation efficiency of VCAM-1 in RNF149 wildtype and knockout cells. To directly detect non-translocated forms of VCAM-1 we used a previously described VCAM-1 that harbors a Flag tag sequence in its signal peptide and is still responsive to CAM741^17^. Flag reactivity directly labels only VCAM-1 forms that have not translocated into the ER lumen where Flag reactivity is lost upon signal peptidase cleavage^26^. Two LMW forms of VCAM-1 that are consistent with lack of glycosylation are detected upon CAM741 and velcade treatment, the higher form that is detected in the HA and Flag immunoblots is the non-translocated pEQC substrate, whereas the lower form that is detected only in the HA immunoblot is an ERAD substrate (Fig. 4A), consistent with deglycosylation of ERAD substrates by p97 cofactor PNGase^27^. Upon quantification of the pEQC non-translocated form of VCAM-1 to the translocated ER form of VCAM-1, we noted an increase in the percentage of the ER form of VCAM-1 in the RNF149 knockout cells upon CAM741 and velcade treatment (Fig. 4B). This result implies that in the absence of RNF149, impaired pEQC processing shifts the balance towards an increase in VCAM-1 translocation. As the balance between the translocation and ubiquitination processes govern the efficiency of translocation, impaired pEQC is expected to tip the balance towards translocation^16^ as observed in the AIRAPL knockout animals^23^. Despite the above, because several pEQC components are also observed in ERAD, our interpretations may not be as conclusive. However, as the AIRAPL-p97-RNF149 complex has a K48 specificity^20^, we assume that ERAD substrates that include K11 ubiquitin linkages^28^, would not be favored by the RNF149-AIRAPL complex. Furthermore, increased ER entry due to pEQC impairment may cause ER stress and ERAD, contributing to the increased ER forms of VCAM-1 observed in the RNF149 KO cells after CAM741 and velcade treatment (Fig. 4B).

### RNF149 regulation as means of tuning quality control

**Fig. 5:**
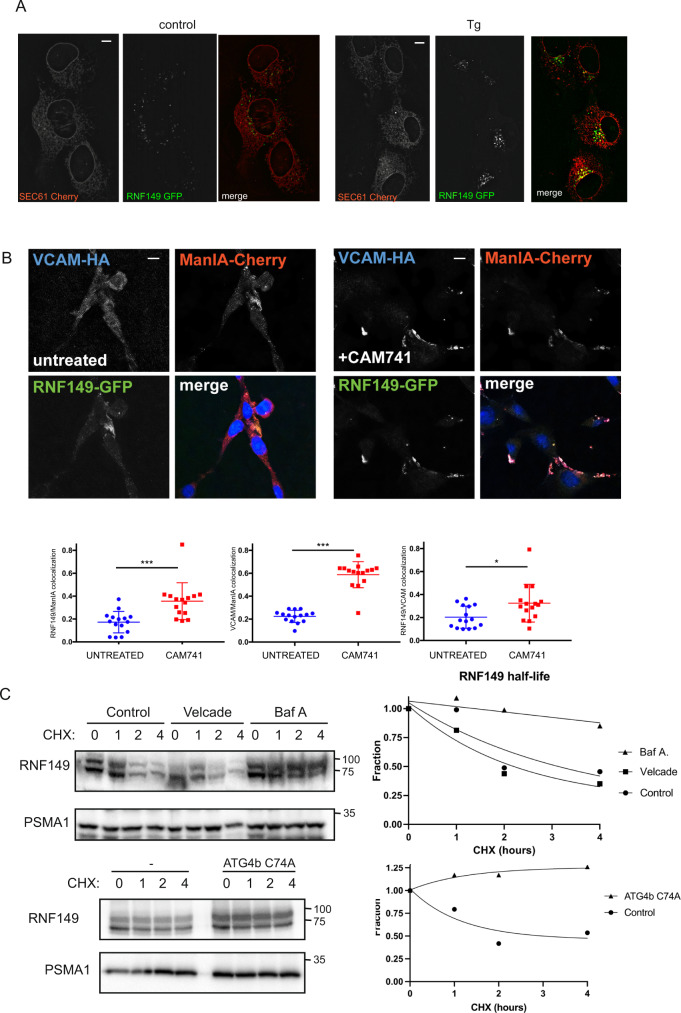
RNF149 regulation as means of tuning quality control. **A** Live cell imaging of cells expressing RNF149-GFP and ER translocon marker Sec61beta-Cherry under control and ER stress conditions. The partial co-localization of RNF149 with Sec61 seems to be induced during ER stress as apparent from the enhanced co-localization (yellow) observed under thapsigargin (Tg) conditions (120 min, 400 nM). Representative images are presented, scale bars are 10um. **B** Immunofluorescence of cells expressing RNF149-GFP, VCAM-HA and ManIA-Cherry show a disperse pattern of VCAM and a partial co-localization of RNF149 and ManIA. Treatment with CAM741 caused accumulation of VCAM at the QCVs, together with ManIA and RNF149. Nuclear staining was performed by DAPI staining and added to the merged figure. Scale bars are 10um. **C** Half-life evaluations of RNF149 were evaluated by treating 293 cells expressing RNF149 with CHX for the indicated time points. The evaluation was performed in the presence or absence of velcade or bafilomycin A (top) or in the presence or absence of a catalytic inactive form of ATG4b (bottom). A quantification of the RNF149 fraction remaining along the four hour time course, is indicated (right).

Translocation regulation is viewed as part of an adaptive mechanism to reduce the folding burden of newly translocating proteins during ER stress^1–3^. In this regard the pEQC can be viewed as an arm of the UPR, however the pEQC components that are regulated by ER stress are not well defined as in the case of various other UPR processes^4–7^. In an attempt to better characterize RNF149 in pEQC stress regulated translocation, we first evaluated the localization of RNF149 using live imaging. RNF149-GFP localization showed a highly dynamic punctate, vesicular pattern that only partially overlapped with the ER and translocon subunit Sec61beta-Cherry. This dynamic vesicular pattern has been previously reported for other PA containing proteins^29^. We next evaluated possible changes in localization during ER stress by treating the cells with Thapsigargin (Fig. 5A). The increased overlap between RNF149 and the Sec61 translocon during ER stress suggests that RNF149 is recruited to the pEQC during ER stress. We have previously reported that class I α-1,2 mannosidase IA (ManIA) is involved in protein quality control and ER-associated degradation and resides in quality control vesicles^30^ (QCVs). ManIA redistributes to juxtanuclear regions during ER stress and is turned over rapidly by autophagic process during steady state conditions^31^. Cell imaging of cells expressing VCAM1-HA, RNF149-GFP and ManIA-Cherry indicate a partial colocalization of RNF149 and ManIA (Fig. 5B). CAM741 treatment causes a strong accumulation of VCAM-HA and significantly increased colocalization with RNF149-GFP and ManIA-Cherry at QCVs, whereas untreated cells showed a weak dispersed pattern of VCAM-1 (Fig. 5B). These results led us to further evaluate if RNF149 is also regulated under steady state conditions by autophagic processes. To this end a cyclohexamide (CHX) time course treatment was performed and samples were evaluated towards RNF149 content. As seen in Fig. 5C, RNF149 was found as a short lived protein with an estimated half-life of 2 h. Its proteolytic disappearance is governed by autophagic rather than proteasomal processes as evident from the stability of the protein under bafilomycin A (BafA) but not velcade treatment conditions. Furthermore, impairment in RNF149 turnover is also observed upon autophagic blockade by means of expression of a catalytic mutant ATG4b.

## Discussion

In this report we identified RNF149 as a new E3 ligase that interacts with various components involved in the proteasomal degradation of mislocalized ER proteins (AIRAPL, p97 and Bag6; Fig. 1). To date RNF149 was reported to be involved in gonocyte development and BRAF induced proliferation^32,33^. Our data presented herein reveal a new role for RNF149 in pEQC processes. While protein quality control process are linked to tumorgenesis, the molecular mechanism linking RNF149 activity to gonocyte development and BRAF induced proliferation remain to be determined. Using two different pEQC substrates (VCAM-1 and IGF1-R), we were able to demonstrate that RNF149 interaction is specific only towards the mislocalized substrate and is not observed when successful translocation is obtained (CAM741 induced mislocalization of VCAM-1 and pro-IGF1-R *vs*. IGF1-R; Fig. 2). Furthermore, the colocalization of VCAM-1 and RNF149 was observed upon CAM741 treatment (Fig. 5). These results highlight the specificity of the RNF149-pEQC substrate interaction that occurs only upon the substrate’s mislocalization and association with factors such as Bag6^15,16^. MS analysis identified the additional interaction of RNF149 with several Sec61 translocon complex subunits as well as numerous EMC components. These finding further emphasize the orchestrated events of the pEQC process that may take place during RQC, ER translocation and ERAD events^34–36^. The involvement of pEQC components such as Bag6 and p97 in several of these protein quality events that occur in proximity to ER membrane components (Translocon and EMC) is probably critical for efficient timing and synchronization between all quality control processes. In-light of this, our finding of Listerin association only with the catalytic active RNF149UBAIT (Fig. 2C) may imply a regulatory step on Listerin that enables synchronization of RQC and pEQC, further exemplifying the interweaving events between protein quality control processes.

The balance between translocation efficiency and proficient processing of pEQC substrates, defines the rate in which substrates can enter the ER^16^. Impairment in pEQC processing tips the balance towards higher translocation and is reported to drive pathological conditions such as MPN^23^. In the case of AIRAPL impairment, this is caused by IGF1-R induced translocation and signaling, which in-turn leads to hematopoietic lineage proliferation^23^. The observed MPN phenotype observed in the RNF149 KO mice is in-accordance at the cellular level with the increased translocation of the examined pEQC substrate (Fig. 3 and Fig. 4). Additionally, tissue gene expression for RNF149 indicate the highest expression found in whole blood samples (Genotype-Tissue Expression Project).

While the adaptive response of the pEQC pathway to ER stress has been reported^1–3^, mechanistic steps in this regulation are still not clear. Highly dynamic vesicular localization that only partially overlaps with the ER membrane may be due to the on-going dynamic surveillance a pEQC E3 ligase continuously performs. Yet the increase of ER localization upon ER stress (Fig. 5), possibly shifts the balance towards higher stringency of ER translocation during ER stress. In-line with this, ER induced accumulation may result from decreased autophagic flux during ER stress^37,38^ and the observed autophagic turn-over of RNF149 may provide a mechanistic explanation to the observed upregulation in pEQC observed during ER stress. This mode of stress induced ER localization has been previously observed in the case of ER-ManI^31^ and our findings demonstrate that recruitment is finely tuned also to pEQC substrate flux, as CAM741 treatment was sufficient even without ER stress to induce colocalization (Fig. 5). Future investigation into the molecular events governing this regulation and the role of the PA luminal domain that governs this localization^29^, will improve our understanding regarding ER stress induced regulation of the pEQC. In this respect, RNF149 may be viewed as a transmembrane sensor that joins a family of ER stress transducers that mediate luminal stress into cytosolic functions that compose the initial sensors and executors of the UPR^4,5,7^.

ER translocation is a complex process that is segmented into various pathways depending on various elements within the newly synthesized protein (signal peptide; transmembrane domain position and tail anchored sequences;^39^). Therefore, general and specific factors dealing with the salvation or degradation of these distinct mislocalized ER proteins are expected to exist. This notion is exemplified nicely in the case of ERAD substrates in which specific and shared factors exist for distinct types of ERAD (ERAD-M, -L, -C^40–42^). Specific cytosolic E3 ligases such as RNF126^43^ would be more suitable in the ubiquitination of mislocalized TA proteins, whereas an ER membrane E3 ligase such as RNF149 maybe better situated towards a co-translationally inserted pEQC substrate. The situation of distinct while partially overlapping ER insertion pathways (SRP vs. SND vs. GET pathway) may also exist in the degradation of mislocalized pEQC substrates that would partially overlap several factors to ensure degradation of MLP. A recent report has placed the cytosolic RNF126 also in the re-ubiquitination of ERAD substrates^44^, thus exemplifying an overlapping process that takes place in order to ensure MLP ubiquitination. Additional factors (such as other E3 ligases or chaperone cofactors that mediate E3 ligase interactions that participate in degradation of MLP’s^45^), could explain in part the mild phenotypes and incomplete inhibition of degradation identified in the case of specific mutations within a specific pEQC E3 ligases such as RNF126^43^ and RNF149 (this report). The involvement of specific pEQC components such as AIRAPL in human MPN pathologies^23^ further emphasizes the importance of establishing in-vivo models for pEQC related pathologies.

## Methods

### Cell culture, lysis, and fractionations

HEK293 cells were cultured in complete medium composed of DMEM supplemented with 1% penicillin–streptomycin solution, 55 μM β-mercaptoethanol, 1% nonessential amino acids solution, and 10% heat-inactivated fetal bovine serum. Where indicated, cells were treated with velcade (100 nM) and/or CAM741 (250 nM) overnight, and cell lysis was performed in TNH buffer (20 mM 4-(2-hydroxyethyl)-1-piperazineethanesulfonic acid (HEPES), pH 7.4, 100 mM NaCl, 1%Triton X-100, 1 mM EDTA, 1.5 mM MgCl2, 1 mM dithiothreitol (DTT), and protease inhibitors) and clarified at 20,000×g for 10 min. Where indicated, cell lysates were directly incubated with Usp-2 as previously described (Baker et al., 2005). For protein half-life chase experiments cells were treated for the indicated time points with cycloheximide (CHX 100ug/ml). 30 min prior to, and throughout the CHX chase, cells were treated with either velcade (10uM) or Bafilomycin A (100 nM) as indicated. Transient transfections were performed using polyethylenimine (PEI) and protein immunopurifications were performed using the indicated antibodies. Immunopurified material was extensively washed with lysis buffer and subsequently eluted with Laemmli buffer. Input material in IP experiments was 50ug while the total lysate content used for the IP was 3-4 mg.

### Cell expression and protein purifications

Plasmids expressing the human AIRAPL, ER-ManI, IGF1R, VCAM-1 or Sec61 were previously described^17,19,23,31^. Mammalian expression plasmids of RNF149 were constructed by amplifying the human RNF149 cDNA and subcloning into a pCDNA3.1 vector in-frame to the indicated tag (Flag or GFP). A predicted RING mutant VW271/299AA was introduced into the relevant expression vector by site-directed mutagenesis (Quikchange, Agilent). A C-terminal ubiquitin fusion of RNF149 (RNF149UBAIT) was constructed as previously demonstrated^25^. For MS analysis, a modified RNF149 WT and mutant Ubait constructs were constructed as previously described^46^. For bacterial expression of RNF149, full length or a cytosolic domain of RNF149 (AA222-400) was subcloned into pET21 (Novagen), overnight auto induction^47^ was performed in BL21 bacteria at 18^0^c and Ni-NTA (GE Healthcare) purifications were performed following manufacturer’s procedure. Quantitative expression of RNF149 transcript was performed using R.T. PCR predesigned primers for mouse RNF149 and HPRT1 genes (Mm01234645 and Mm00446968) according to manufacturer conditions (Applied Biosystems). All experiments were performed in triplicates.

### Mass-spectrometry, data processing and analysis

RNF149Ubait elutions were supplemented with 5% SDS in 50 mM Tris-HCl. The protein was reduced with 5 mM dithiothreitol and alkylated with 10 mM iodoacetamide in the dark. Each sample was loaded onto S-Trap microcolumns (Protifi, USA) trypsin digested according to the manufacturer’s instructions. Each sample was loaded using split-less nano-Ultra Performance Liquid Chromatography (10 kpsi nanoAcquity; Waters, Milford, MA, USA). Desalting of the samples was performed online using a reversed-phase Symmetry C18 trapping column (Waters) and peptides separated using a T3 HSS nano-column (Waters). Peptides were eluted from the column into the quadrupole orbitrap mass spectrometer (Q Exactive Plus, Thermo Scientific) using a FlexIon nanospray apparatus (Proxeon) and data acquired in data dependent acquisition (DDA) mode, using a Top10 method. Data processing and analysis was performed using Maxquant software using the default parameters. Data was searched against the human sequences Swissprot appended with common laboratory contaminant proteins and the relevant RNF149 sequence. Search results were filtered to achieve maximum false discovery rate of 1% at the protein level. LFQ intensities were used for further calculations using Perseus version 1.6.2.3. Decoy hits were filtered out, as well as proteins that were identified on the basis of a modified peptide only. Intensities were log transformed and only proteins that had at least 2 valid values in at least one experimental group were kept. The remaining missing values were imputed. A Student’s *t*-test was used to identify significant differences across the biological replica. Fold changes were calculated based on the ratio of geometric means of the WT versus mutant samples.

### In-Vitro Ubiquitination

Enzymatic activity of cytosolic recombinant RNF149 was performed by incubating the recombinant expressed ligase (WT or RING mutant) with ubiquitin (SigmaU6253), E1 (produced as previously described^48^), and UbcH5b^49^. Reactions were performed at 25^0^c in activity buffer (50 mM Tris 7.5, 5 mM MgCl_2_, 2 mM ATP, 10 mM creatine phosphate, 0.1 mg/ml creatine kinase, 0.1 mM 2-mercaptoethanol) for the indicated time. Self-ubiquitination was evaluated by immunoblots against RNF149 and evaluation of RNF149 increase in SDS-PAGE migration. E2 and active RING dependency were performed by omitting or substituting in the reaction as indicated.

### Antibodies and western blots

AIRAP, AIRAPL, GFP and p97 antibodies were produced as previously described^21,50,51^. RNF149 antiserum was produced by immunizing rabbits against full length RNF149. The sources for the following antibodies were: Anti-Flag (M2 sigma-aldrich), HA (16B12 Covance), Bag6 (Cell signaling), RNF149 (Origene TA810580), PSMA1 (a kind gift from Keiji Tanaka), Rpt6 (a kind gift from Shigeo Murata) and Actin monoclonal antibodies were used as loading controls for immunoblots.

### Generation of RNF149 knockout and analyses

RNF149 knockout mice were generated using CRISPR/Cas9 gene targeting technology as described^52^. Two crRNA’s were designed to target exon1 and exon5 of RNF149 (CCGGGACGCACTCGCGGTGC & GTTTACACATTGGACACGTT-pair1 or ACACCGTCAGGTTCGACTGC & CGTAACAAACCCAATATCCC-pair2). In-vitro transcribed Cas9 RNA(100 ng/ul), and sgRNA (EnGen® sgRNA Synthesis Kit, 50 ng/ul), were injected into the cytoplasm of one-cell fertilized embryos isolated from super ovulated CB6F1 hybrid mice mated with CB6F1 males (Harlan Biotech Israel Ltd. Rehovot, Israel). Injected embryos were transferred into the oviducts of pseudopregnant ICR females. Genomic DNA from resulting pups was analyzed at weaning for the genomic deletion by PCR and Sanger sequencing using primers from exon1 and exon5 (475-TGGAGTGAGTCGCCTCGTTC; 492-CTTAGACAAGTAGGTGGACTTCATG; 477-TTTCATCTGTTCACCTTGCC). All mouse experiments were approved by the Weizmann Institute’s IACUC committee and were carried out in accordance with their approved guidelines. Mouse fibroblasts were produced from day 13.5 embryos. Hematological analyses were performed using a Abaxis Vetscan HM5 (according to the manufacturer’s procedure) on 6 month old animals.

### Fluorescence microscopy

Live images of RNF149-GFP and Sec61-Cherry were acquired from U2OS transfected cells. Cells were grown on 35 mm glass-bottom dishes (SPL life science). At 24 h post transfection, images were acquired in an wide field Olympus IX83 microscope (Olympus, Japan) equipped with ×100 oil immersion objective Plan Apo100x oil (na1.45 wd 0.13 mm), Orca Flash 4.0 camera (Hamamatsu Photonics, Japan) and a LED light source (CoolLED, UK). Cells were kept in a stage incubator which provides tissue culture conditions (37 °C, 5% CO2). Images were acquired using cellsens software and processed using the online deblur tool. Immunofluorescence microscopy of NIH 3T3 cells transfected with VCAM1-HA, RNF149-GFP and ManIA-Cherry, treated and grown on glass coverslips. Cells were incubated overnight with or without 250 nM of CAM741. At 24 h post-transfection, the cells were rinsed twice with PBS, fixed with 3% Paraformaldehyde for 30 min at room temperature, then incubated for 5 min with 50 mM Glycine and permeabilized with 0.5% Triton X-100 for 10 min. After blocking with 50 µg/ml normal goat IgG in PBS/BSA 2%, the cells were incubated with mouse anti-HA primary antibody diluted according to the manufacturer’s recommendation, washed 3 times with PBS/BSA 2% and incubated for 30 min with goat anti-mouse IgG-dylight 649 diluted according to the manufacturer’s recommendation, followed by nuclear staining with DAPI (Sigma). The coverslips were then mounted on slides and examined using a Zeiss LSM 510 Meta confocal microscope.

### Statistics and reproducibility

Hematological statistical analyses were performed using a two tailed Mann-Whitney test on the data obtained using a Abaxis Vetscan HM5. Statistical evaluations of VCAM-1, ManIA and RNF149 colocalizations were performed using *t*-test evaluation on data obtained from the Zeiss LSM 510 Meta confocal microscope.

### Reporting summary

Further information on research design is available in the Nature Portfolio Reporting Summary linked to this article.

## Supplementary information

Supplementary Information

Reporting Summary

## Data Availability

The mass-spectrometry datasets generated during the current study are available in the PRIDE repository partner repository with the dataset identifier PXD038478 and 10.6019/PXD038478. Uncropped blots are available as Supplementary Fig. 2.
