# Supplementary material for: The role of RNF149 in the pre-emptive quality control substrate ubiquitination
Source: Commun Biol. 2023 Apr 8;6:385. doi: 10.1038/s42003-023-04763-9 (PMC10082771; doi:10.1038/s42003-023-04763-9)
Supplement: Supplementary file 1 — Supplementary Information [file 42003_2023_4763_MOESM1_ESM.pdf]

Supplemental Figure 1

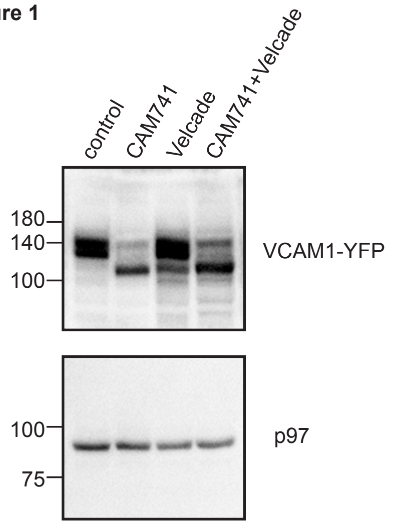

Supplemental figure 1. pEQC reporter cell line
293 stably expressing VCAM1-YFP were treated with CAM741 or velcade as indicated in-order to confirm the correct processing of the fusion protein by the pEQC. VCAM1-YFP content was evaluated by immunoblot.

Supplementary Figure 2

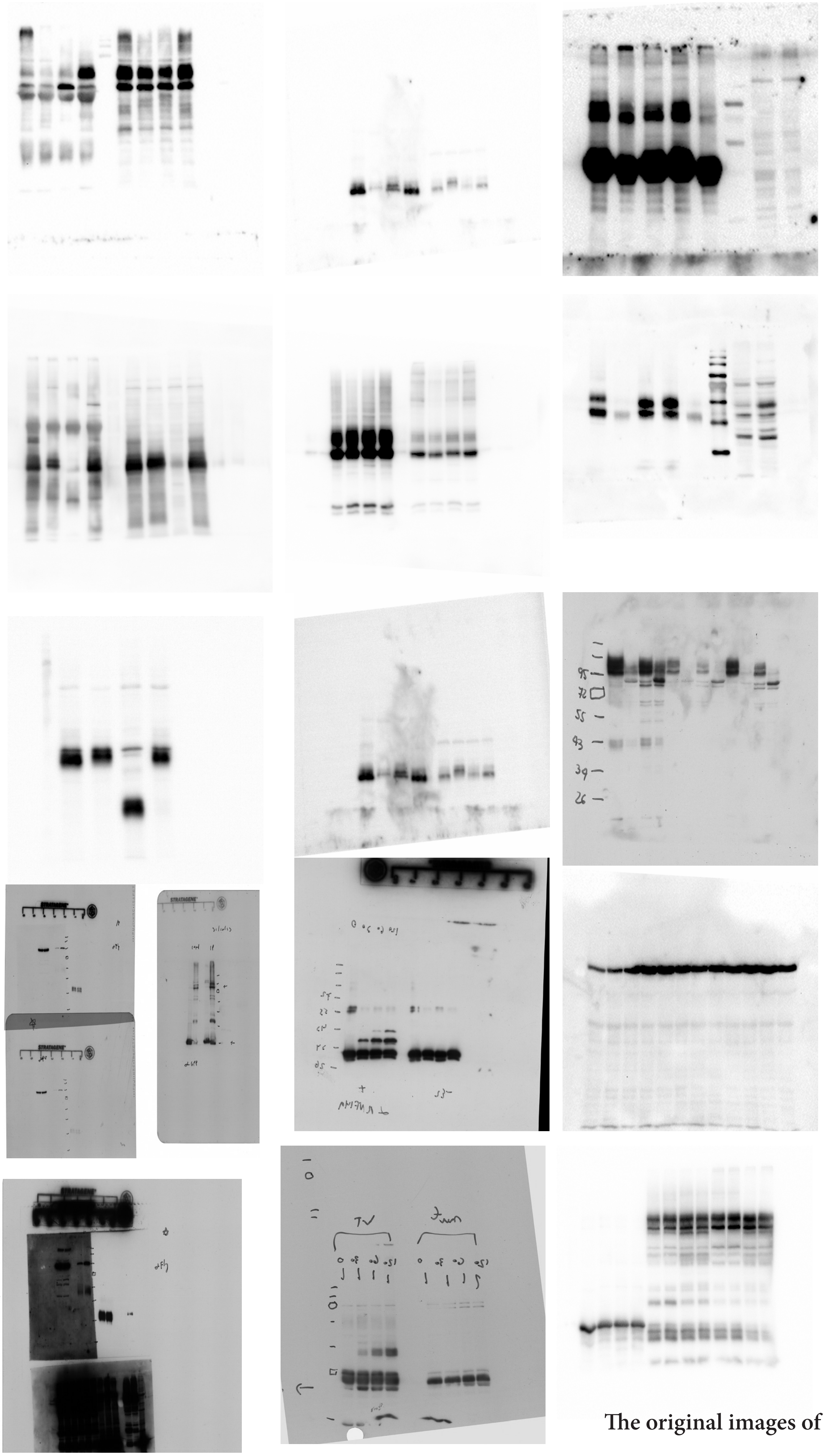

The original images of blots of Fig. 1

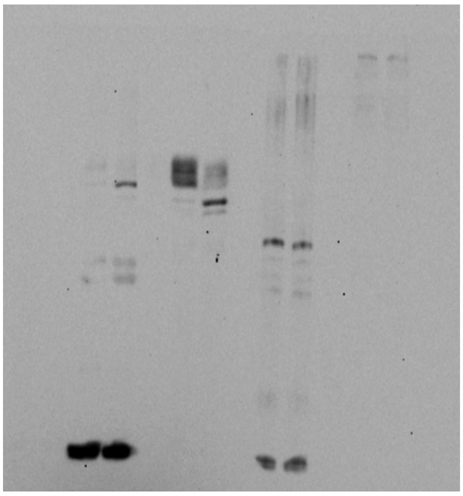

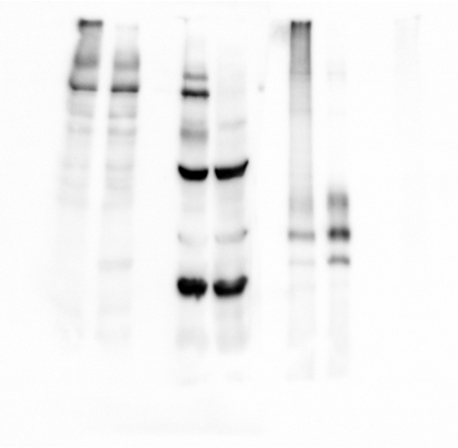

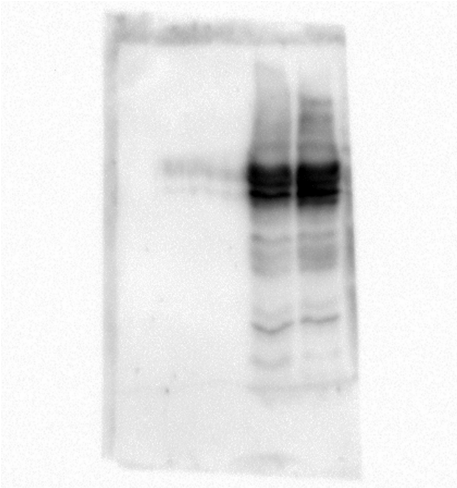

The original images of blots of Fig. 2

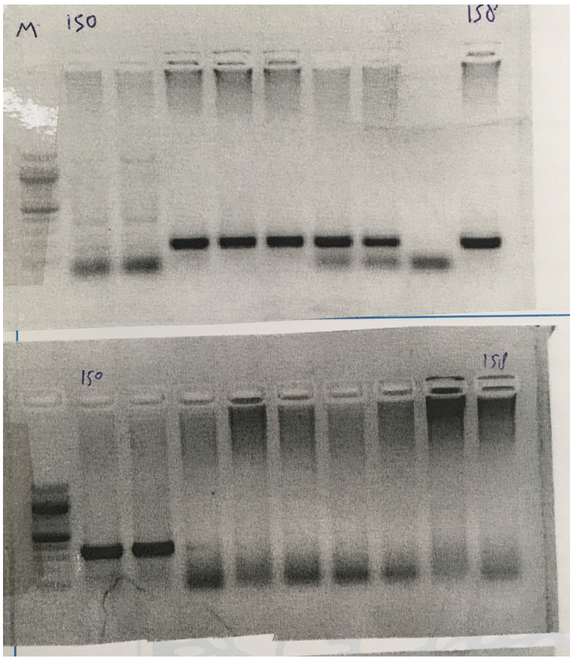

The original images of gels of Fig. 3

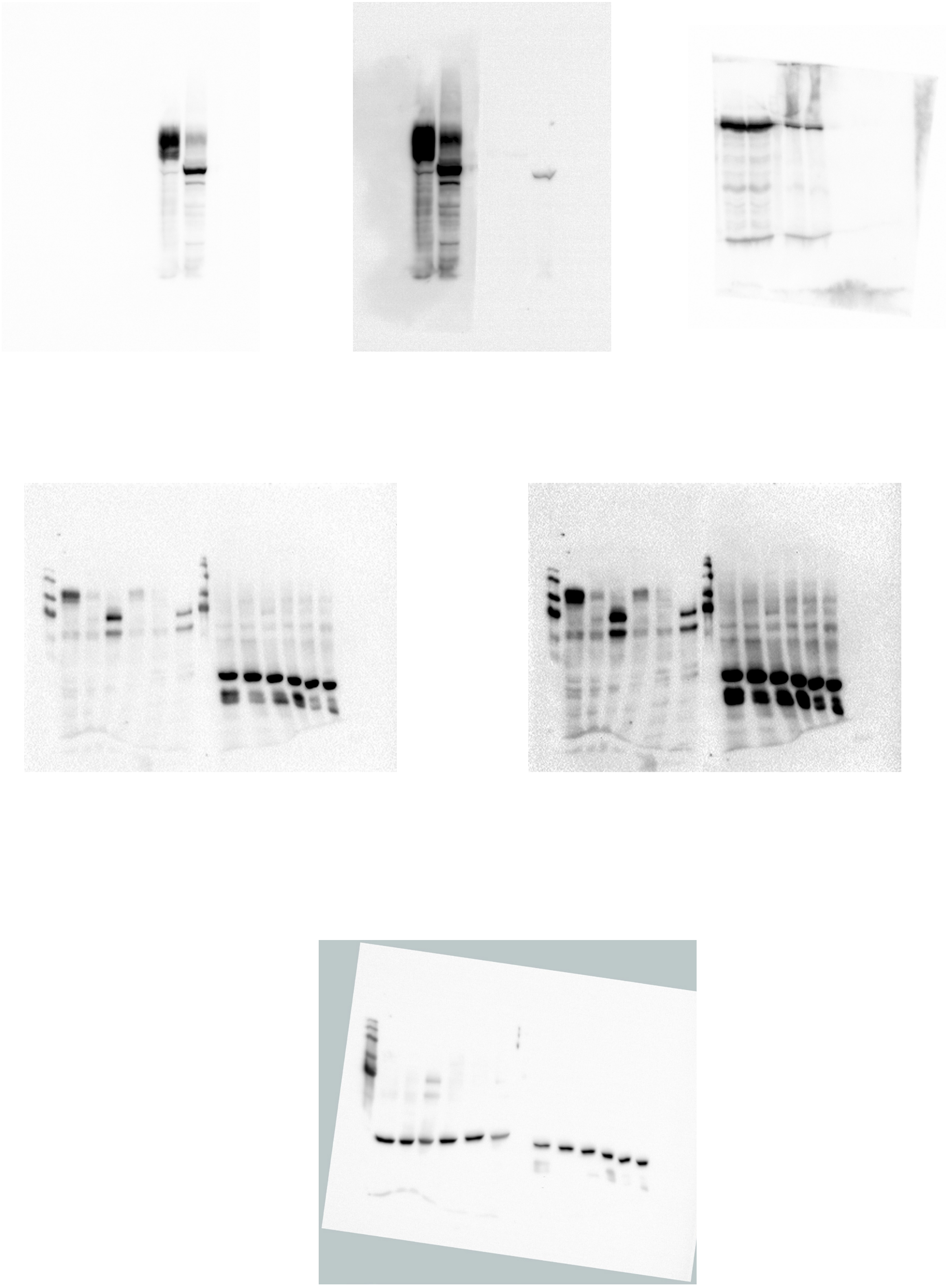

The original images of blots of Fig. 4

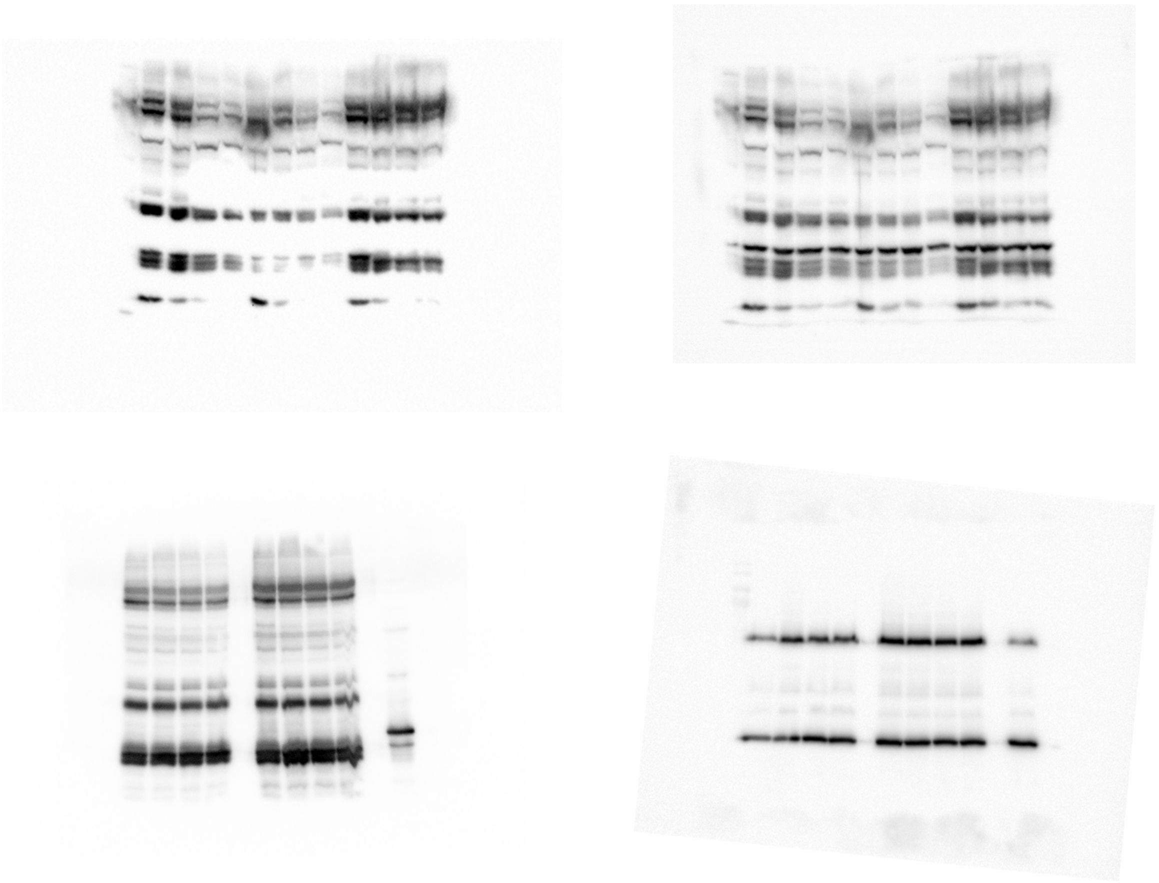

The original images of blots of Fig. 5

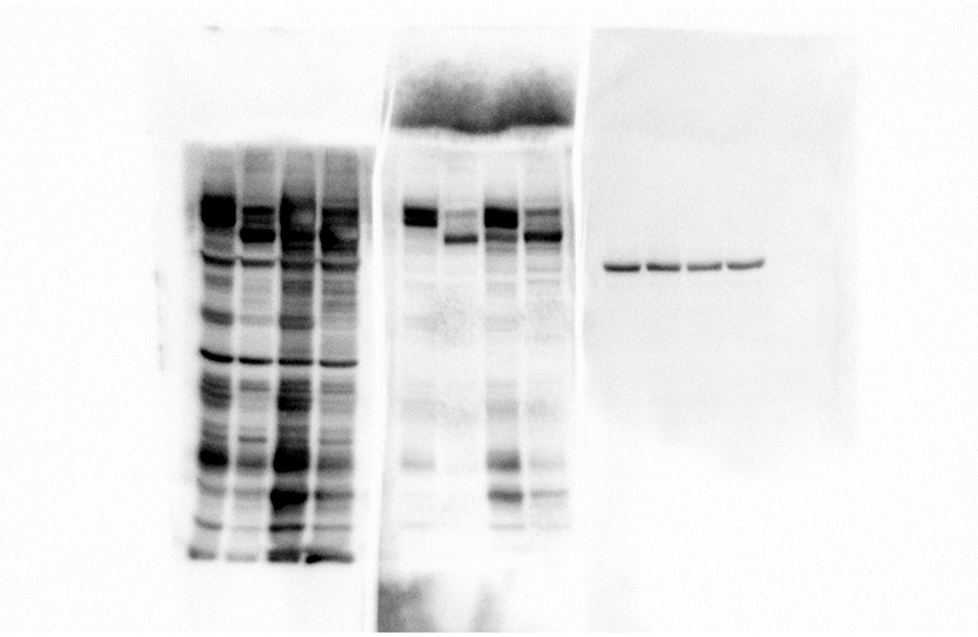

The original images of blots of Fig. S1
