# Supplementary material for: The role of RNF149 in the pre-emptive quality control substrate ubiquitination
Source: Commun Biol. 2023 Apr 8;6:385. doi: 10.1038/s42003-023-04763-9 (PMC10082771; doi:10.1038/s42003-023-04763-9)
Supplement: Supplementary file 2 — Reporting Summary [file 42003_2023_4763_MOESM2_ESM.pdf]

nature portfolio

Corresponding author(s): *Double-blind peer review submissions: write DBPR and your manuscript number here instead of author names.*

Last updated by author(s): *YYYY-MM-DD*

Reporting Summary

Nature Portfolio wishes to improve the reproducibility of the work that we publish. This form provides structure for consistency and transparency in reporting. For further information on Nature Portfolio policies, see our Editorial Policies and the Editorial Policy Checklist.

Statistics

For all statistical analyses, confirm that the following items are present in the figure legend, table legend, main text, or Methods section.

n/a	Confirmed	
☐	☒	The exact sample size (n) for each experimental group/condition, given as a discrete number and unit of measurement
☐	☒	A statement on whether measurements were taken from distinct samples or whether the same sample was measured repeatedly
☐	☒	The statistical test(s) used AND whether they are one- or two-sided<br>*Only common tests should be described solely by name; describe more complex techniques in the Methods section.*
☒	☐	A description of all covariates tested
☐	☒	A description of any assumptions or corrections, such as tests of normality and adjustment for multiple comparisons
☐	☒	A full description of the statistical parameters including central tendency (e.g. means) or other basic estimates (e.g. regression coefficient) AND variation (e.g. standard deviation) or associated estimates of uncertainty (e.g. confidence intervals)
☐	☒	For null hypothesis testing, the test statistic (e.g. F, t, r) with confidence intervals, effect sizes, degrees of freedom and P value noted<br>*Give P values as exact values whenever suitable.*
☒	☐	For Bayesian analysis, information on the choice of priors and Markov chain Monte Carlo settings
☒	☐	For hierarchical and complex designs, identification of the appropriate level for tests and full reporting of outcomes
☒	☐	Estimates of effect sizes (e.g. Cohen's d, Pearson's r), indicating how they were calculated

Our web collection on statistics for biologists contains articles on many of the points above.

Software and code

Policy information about availability of computer code

Data collection	Data collection of the specified images from microscopy experiments was performed using the manufactures provided Images were acquired using cellsens software software
Data analysis	Immunoblot presentation and annotation using adobe indesign, text using microsoft word, graph and statistics using Graphpad prism and bibliography using Eendnote.

For manuscripts utilizing custom algorithms or software that are central to the research but not yet described in published literature, software must be made available to editors and reviewers. We strongly encourage code deposition in a community repository (e.g. GitHub). See the Nature Portfolio guidelines for submitting code & software for further information.

Data

Policy information about availability of data

All manuscripts must include a data availability statement. This statement should provide the following information, where applicable:

- Accession codes, unique identifiers, or web links for publicly available datasets
- A description of any restrictions on data availability
- For clinical datasets or third party data, please ensure that the statement adheres to our policy

web links for publicly available data have been provided

Human research participants

Policy information about studies involving human research participants and Sex and Gender in Research.

Reporting on sex and gender	NA
Population characteristics	*Describe the covariate-relevant population characteristics of the human research participants (e.g. age, genotypic information, past and current diagnosis and treatment categories). If you filled out the behavioural & social sciences study design questions and have nothing to add here, write "See above."*
Recruitment	*Describe how participants were recruited. Outline any potential self-selection bias or other biases that may be present and how these are likely to impact results.*
Ethics oversight	*Identify the organization(s) that approved the study protocol.*

Note that full information on the approval of the study protocol must also be provided in the manuscript.

Field-specific reporting

Please select the one below that is the best fit for your research. If you are not sure, read the appropriate sections before making your selection.

☒ Life sciences ☐ Behavioural & social sciences ☐ Ecological, evolutionary & environmental sciences

For a reference copy of the document with all sections, see nature.com/documents/nr-reporting-summary-flat.pdf

Life sciences study design

All studies must disclose on these points even when the disclosure is negative.

Sample size	we used all animals born that were set aside for MPN evaluations
Data exclusions	No data was excluded
Replication	all replications were successful
Randomization	Animals set aside for MPN evaluations were randomly picked from colony and set aside during early age
Blinding	Blinding sample aquisition was not applicable during animal care as all mice cages are labeled.

Reporting for specific materials, systems and methods

We require information from authors about some types of materials, experimental systems and methods used in many studies. Here, indicate whether each material, system or method listed is relevant to your study. If you are not sure if a list item applies to your research, read the appropriate section before selecting a response.

Materials & experimental systems

n/a	Involved in the study	
☐	☒	Antibodies
☐	☒	Eukaryotic cell lines
☒	☐	Palaeontology and archaeology
☐	☒	Animals and other organisms
☒	☐	Clinical data
☒	☐	Dual use research of concern

Methods

n/a	Involved in the study	
☒	☐	ChIP-seq
☒	☐	Flow cytometry
☒	☐	MRI-based neuroimaging

Antibodies

Antibodies used	AIRAP, AIRAPL, GFP and p97 antibodies were produced as previously described (see ref. 21,50, 51). RNF149 antiserum was produced by immunizing rabbits against full length RNF149. The sources for the following antibodies were: Anti-Flag (M2 sigma-aldrich), HA (16B12 Covance), Bag6 (Cell signaling), RNF149 (Origene TA810580), PSMA1 (a kind gift from Keiji Tanaka), Rpt6 (a kind gift from Shigeo Murata) and Actin monoclonal antibodies were used as loading controls for immunoblots.

Validation	To the exception of the RNF149 polyclonal antibody we produced, all antibobies sources and references anre described in the methodes section inder section antibodies and western blots. The RNF149 antibody producded was validated by using lysates of 293 cells over-expressing the human RNF149 for immunoblots and immunopercipitation.

Eukaryotic cell lines

Policy information about cell lines and Sex and Gender in Research

Cell line source(s)	U2OS (ATCC HTB-96), HEK293 (ATCC CRL1573), NIH3T3 (ATCC CRL1658), Mouse embryonic fibroblasts were produced from the indicated RNF149 genotype mice at embryonic day 13.5.
Authentication	none of the cell lines was authenticated
Mycoplasma contamination	All lines were tested and found negative for mycoplasma
Commonly misidentified lines (See ICLAC register)	*Name any commonly misidentified cell lines used in the study and provide a rationale for their use.*

Animals and other research organisms

Policy information about studies involving animals; ARRIVE guidelines recommended for reporting animal research, and Sex and Gender in Research

Laboratory animals	C57Bl6 mice and the indicaed age of 6months
Wild animals	the study did not involve wild animals
Reporting on sex	findings do not apply only to one sex
Field-collected samples	the study did not involve samples collected from the field
Ethics oversight	All mouse experiments were approved by the Weizmann Institute of Science institutional animal care and use committee (IACUC) and were carried out in accordance with their approved guidelines

Note that full information on the approval of the study protocol must also be provided in the manuscript.
